# Supplementary material for: MetaRibo-Seq measures translation in microbiomes
Source: Nat Commun. 2020 Jun 29;11:3268. doi: 10.1038/s41467-020-17081-z (PMC7324362; doi:10.1038/s41467-020-17081-z)
Supplement: Supplementary file 10 — Supplementary Data 7 [file 41467_2020_17081_MOESM10_ESM.zip › File2/Confidence_High_Taxonomy/108600_out.krona.html]

Javascript must be enabled to view this page.

members
magnitude
magnitudeUnassigned
count
unassigned
taxon
rank

108600\_out

20

superkingdom
2
20

phylum
976
20

class
200643
20

order
171549
20

family
815
20

genus
20
816

1
46506

SRS142599\_contig\_number\_15831
species


SRS056273\_contig\_number\_449
species
1
1929999

species

SRS075078\_contig\_number\_30272
818
1

species

SRS144537\_contig\_number\_49143
246787
1

817
1
species

SRS104636\_contig\_number\_15705

1263049
2
species

SRS019286\_contig\_number\_6125SRS147139\_contig\_number\_31192

13
329854

SRS011405\_contig\_number\_contig-100\_1478.114020SRS012273\_contig\_number\_11948SRS016629\_contig\_number\_contig-100\_571.34973SRS019445\_contig\_number\_37268SRS020869\_contig\_number\_10662SRS049995\_contig\_number\_10325SRS076756\_contig\_number\_22216SRS076976\_contig\_number\_21599SRS1041137\_contig\_number\_14326SRS1055043\_contig\_number\_19663SRS1055099\_contig\_number\_10824SRS147346\_contig\_number\_49666SRS893369\_contig\_number\_12877
species
